# 3D Breast Cancer Spheroids Reveal Architecture-Dependent HER2 Expression and Signaling

**DOI:** 10.3390/biology14121654

**Published:** 2025-11-24

**Authors:** Pietro Arnaldi, Valentina Delli Zotti, Grazia Bellese, Maria Cristina Gagliani, Paola Orecchia, Patrizio Castagnola, Katia Cortese

**Affiliations:** 1 Department of Experimental Medicine (DIMES), MorphoLAB, University of Genoa, 16132 Genoa, Italy; pietroarnaldi.bio@gmail.com (P.A.); bgrazia@unige.it (G.B.); gagliani@unige.it (M.C.G.); 2 IRCCS Ospedale Policlinico San Martino, 16132 Genova, Italy; paola.orecchia@hsanmartino.it (P.O.); patrizio.castagnola@gmail.com (P.C.); 3 Department of Experimental Medicine (DIMES), University of Genoa, 16132 Genoa, Italy; 4491466@studenti.unige.it

**Keywords:** HER2-positive breast cancer, HER2/ERBB2, 3D cell culture, tumor spheroids, tissue architecture, confocal microscopy, electron microscopy, cell–cell interactions

## Abstract

Background: Three-dimensional (3D) culture systems offer a physiologically relevant alternative to monolayers for studying tumor organization, signaling, and drug response. HER2-positive breast cancers (BCa) account for 15–30% of BCa cases and benefit from HER2-targeted therapies, yet predictive in vitro models remain limited. Objective: To generate and compare 3D spheroids from two HER2+ BCa cell lines, SKBR3 and BT474, and investigate how 3D architecture influences HER2 distribution, intracellular signaling, and cellular organization. Methods: Spheroids were reproducibly generated from SKBR3 and BT474 cells and analyzed after 4 days of culture. Cell viability was evaluated using live/dead staining, HER2 distribution was assessed by confocal microscopy and quantified on cryosections, and protein expression/phosphorylation was measured by Western blotting. Epithelial and EMT markers were visualized by immunofluorescence, and ultrastructural features were examined by transmission electron microscopy (TEM). Results: Both cell lines formed viable spheroids with distinct architectures: SKBR3 spheroids were loose and heterogeneous, whereas BT474 spheroids were compact and highly spherical. Confocal and cryosection imaging showed consistent membrane HER2 localization with a progressive signal decrease toward the core of the spheroids, more pronounced in BT474. Western blotting revealed divergent HER2 expression and AKT phosphorylation: SKBR3 spheroids displayed increased HER2 but reduced pAKT, while BT474 spheroids showed reduced HER2 and pAKT levels. EpCAM and E-cadherin staining revealed cell line-specific epithelial organization, and TEM demonstrated differences in intercellular spacing and mitochondrial morphology, reflecting spheroid compactness. Conclusions: 3D architecture profoundly influences HER2 distribution, signaling, and structural organization in HER2+ BCa spheroids. This model provides a robust platform for investigating architecture-dependent molecular processes, with potential applications in drug response, receptor trafficking, and targeted therapy evaluation.

## Simple Summary

Breast cancer is a highly heterogeneous disease, and about 15–30% of cases are driven by the HER2 receptor, a protein that can be specifically targeted by modern therapies. Traditionally, cancer cells are grown in flat layers on plastic, but this does not reproduce the complexity of a real tumor. Three-dimensional (3D) culture systems, where cells grow as spherical clusters called “spheroids,” provide a more realistic environment that mimics how cells interact in the body. In this study, we generated 3D spheroids from two commonly used HER2-positive breast cancer cell lines, SKBR3 and BT474. Although both cell types formed viable spheroids, their shapes and internal organization were very different: SKBR3 spheroids were loose and irregular, while BT474 spheroids were compact and highly spherical. We examined how HER2 was distributed within these structures and found that its signal decreased toward the spheroid core, especially in BT474. We also analyzed signaling proteins and cellular structures, discovering differences in cell–cell adhesion, mitochondrial features, and key molecular pathways. Overall, our results show that the 3D architecture of breast cancer spheroids strongly influences how cells organize, signal, and maintain HER2 expression. This system provides a powerful tool to study drug responses and to improve strategies for personalized therapies.

## 1. Introduction

Breast cancer (BCa) remains the most frequently diagnosed malignancy and a leading cause of cancer-related death among women worldwide, accounting for over 2.3 million new cases annually [1]. Among molecular subtypes, HER2/ERBB2-positive tumors represent approximately 15–30% of cases and are characterized by aggressive clinical behavior but susceptibility to HER2-targeted therapies, including monoclonal antibodies such as trastuzumab and, more recently, antibody–drug conjugates (ADCs) like trastuzumab–deruxtecan (T-DXd) [2,3,4]. Despite these advances, intrinsic and acquired resistance remain major clinical challenges, partly reflecting the limited predictive power of conventional two-dimensional (2D) monolayer cultures to reproduce the spatial and biochemical complexity of solid tumors [5,6]. Three-dimensional (3D) culture models, including multicellular spheroids, offer a more physiologically relevant platform that recapitulates key features of the tumor microenvironment, such as cell–cell and cell–matrix interactions, nutrient and oxygen gradients, and regional heterogeneity in proliferation and signaling [7,8,9]. Importantly, 3D organization can modulate receptor accessibility, endocytic trafficking, and downstream signaling, parameters that are particularly critical for the efficacy of ADCs and other targeted therapies [8,10,11,12]. Previous studies have shown that HER2+ BCa cell lines display markedly different morphologies and growth behaviors in 3D culture: BT474 cells form highly compact spheroids, whereas SKBR3 cells generate looser, grape-like aggregates [9,11]. However, most available reports focus primarily on drug sensitivity or viability endpoints, with limited integration of morphological, ultrastructural, and biochemical profiling.

Here, we systematically characterized and compared spheroids derived from SKBR3 and BT474 cells using a multimodal approach including live/dead viability assays, confocal microscopy, immunofluorescence on cryosections, transmission electron microscopy (TEM), and Western blotting. Unlike most previous studies, which have focused mainly on drug sensitivity or gross viability endpoints, we provide an integrated spatial and biochemical profiling of HER2 in 3D, quantitatively mapping its peripheral-to-core gradients and linking them to downstream activation (pHER2/pAKT) in two distinct HER2+ BCa models. We further relate epithelial adhesion and mesenchymal markers (EpCAM, E-cadherins, N-cadherins) to spheroid compactness and highlight early mitochondrial remodeling as a structural correlate of 3D adaptation. By integrating morphological, ultrastructural, and signaling data, this study establishes a reproducible agarose-microwell platform for investigating architecture-dependent receptor regulation and offers a robust platform for future preclinical studies on drug response and targeted delivery in HER2+ breast cancer.

## 2. Materials and Methods

### 2.1. Cell Culture

ERBB2+ overexpressing BCa cell lines SKBR-3 and BT474 were obtained from Banca Biologica and Cell Factory in IRCCS Ospedale Policlinico San Martino belonging to the European Culture Collection’s Organization (Porton Down, Wiltshire, UK). Both cell lines were cultured in DMEM high-glucose medium supplemented with 10% heat-inactivated fetal bovine serum, 1% glutamine, penicillin, and streptomycin (Euroclone S.p.A., Milan, Italy). Two-dimensional cell cultures were conducted by cell seeding at 100,000 cells/well in 6-well plates or onto glass coverslip for imaging. Spheroids were made using a non-adherent agarose substrate, with U-shaped wells with a diameter of 550 µm, in each of which ~20,000 cells were seeded. The substrate was created using the replica-molding technique by 3D printing the negative cast, into which a hot agarose solution (1% *w*/*v*) was poured, allowed to solidify, and subsequently removed and sterilized by exposure to UV light [13,14]. Samples were kept inside the well for the entire culture time, replacing 75% of the culture medium every 2 days.

### 2.2. Optical Microscopy

Brightfield optical microscopy (Zeiss Axio Imager M1, Carl Zeiss Industrielle Messtechnik GmbH, Oberkochen, Germany) was employed for daily monitoring of spheroid development. The growth profile over time in culture of the samples in terms of diameter and circularity was evaluated using 1.47p version of ImageJ software (ImageJ, U. S. National Institutes of Health, Bethesda, MD, USA). The viability of the spheroids was assessed by live/dead staining (NUCLEAR-ID^®^ Blue/Red cell viability reagent—GFP-CERTIFIED, Enzo Life Science, Farmingdale, NY, USA) on the last day in culture (DIV4). Spheroids were stained and incubated for 45 min before being observed under the microscope; consequently, samples were disassembled into single cells by trypsinization to perform a numerical assessment of viability. Brightfield and live/dead fluorescent images acquisition was performed using an Olympus IX70 (Olympus Corporation, Tokyo, Japan) widefield microscope equipped with Hamamatsu camera Orca-Flash 4.0 V3/LT+ (Hamamatsu, Japan) and 10× and 20× non-immersion objectives. Full-thickness spheroids immunostaining was performed by fixing cells in 3.3% paraformaldehyde (PFA) in phosphate-buffered saline (PBS), pH 7.4, for 40 min and then quenching with 30 mM NH_4_Cl. Cell permeabilization was performed by exposure for 10 min to a PBS diluted solution of Saponin from Quillaja Bark (0.2% *w*/*v*, Sigma-Aldrich, 3050 Spruce Street, St. Louis, MO 63103, USA). Afterwards, samples were incubated with an Alexa Fluor 594 Phalloidin (Thermofisher Scientific, Waltham, MA, USA) and mouse anti-ErbB2 9G6 (Santa Cruz Biotechnology, Dallas, TX, USA), both diluted 1:400 in 0.1% *w/v* Saponin solution for 40 min. Subsequently, cells were washed 3 times with PBS and incubated with secondary antibody conjugated to Alexa Fluor 488 (Thermofisher Scientific, Waltham, MA, USA) and DAPI for additional 40 min. Finally, samples were washed with PBS, transferred to a bottom glass Petri dish (3 cm) and observed with Leica DMi8 widefield microscope equipped with 20× non-immersion objective (Leica Microsystems GmbH. Ernst-Leitz-Strasse 17-37. 35578 Wetzlar Germany). Images were processed with the Leica Thunder computational clearing method (LAS X).

### 2.3. Immunofluorescence and Histological Analysis of Spheroids Formed by HER2+ BCa Cell Lines

To prepare cryosections, spheroids were washed in PBS, fixed with 4% PFA for 1 h at 4 °C, resuspended in Bio-agar (Bio-Optica, Milano, Italy), and, after a further 3 h fixation in 4% PFA, they were dehydrated with 30% (*w*/*v*) sucrose overnight (ON). The next day samples were frozen in O.C.T. Compound (Bio-Optica) and were stored at −80 °C. Then, 6 µm thick cryo-sections were cut, dried ON at room temperature (RT), and used for histological examination, using hematoxylin, and immunofluorescence characterization. For immunofluorescence analysis, spheroid cryo-sections were blocked and permeabilized in 0.5% FCS (fetal calf serum) and 0.1% Triton X-100 in PBS for 1 h RT. Then, sections were incubated with the following primary antibodies at RT for 1 h: anti-Ki-67 (2 µg/mL, clone 30.9, Ventana Medical Systems, Inc., Tucson, AZ, USA), anti-E-cadherin (5 µg/mL, 67A4, Santa Cruz Biotechnology, Dallas, TX, USA), anti-N-cadherin (10 µg/mL, 8C11, Santa Cruz), anti-HER2 (0.5 µg/mL, 9G6, Santa Cruz), anti-EpCAM (5 µg/mL, HEA125, Santa Cruz). After 30 min of incubation with Alexa-Fluor-conjugated isotype-specific secondary antibody at RT, sections were mounted with ProLong™ Diamond Antifade Mountant containing DAPI (Thermo Fisher Scientific, Waltham, MA, USA) for nuclear staining. Digital images were acquired using the Aperio VERSA Brightfield, Fluorescence & FISH Digital Pathology Scanner (Leica Biosystems, Milano, Italy), with a 20× or a 40× objective.

### 2.4. TEM

For ultrastructural analysis, SKBR3 and BT474 cells were washed twice in 0.1 M cacodylate buffer (pH 7.4, Sigma-Aldrich, 3050 Spruce Street, St. Louis, MO 63103, USA) and fixed in 0.1 M cacodylate buffer containing 2.5% glutaraldehyde (Electron Microscopy Science, Hatfield, PA, USA) for 1 h at room temperature. The cells were postfixed in 1% osmium tetroxide for 10 min (VWR International, One Radnor Corporate Center, 100 Matsonford Road, Radnor, PA 19087, USA) and 1% aqueous uranyl acetate (SERVA Electrophoresis GmbH, Heidelberg, Germany) for 1 h at room temperature in the dark. Subsequently, samples were dehydrated through a graded ethanol series (70%, 90%, 100%; 5 min each) (Merck, Darmstadt, Germany) and flat-embedded in epoxy resin (Poly/Bed 812; Polysciences Inc., Warrington, PA, USA). Polymerization was carried out for 24 h at 60 °C. Ultrathin sections (50 nm) were cut parallel to the cell monolayer and counterstained with 5% uranyl acetate in 50% ethanol. Ultrathin sections (~50 nm) were cut using a diamond knife on an ultramicrotome (Leica UCT, Leica Microsystems, Vienna, Austria), mounted on copper grids, and counterstained with 5% uranyl acetate in 50% ethanol and Reynolds’ lead citrate. Images were acquired with a Hitachi HT7800 transmission electron microscope (120 kV; Hitachi, Tokyo, Japan) equipped with a Megaview G3 digital camera and Radius 2.0 software (EMSIS, Münster, Germany). For EM morphometry, twenty randomly selected cells per condition were analyzed for mitochondrial number and area using the line tool in Radius 2.0 (*n* = 3 independent experiments) [15]. For EM morphometry, 20 whole cells were scored for mitochondria and measured with line tool of Radius 2.0 software (N = 3 independent experiments).

### 2.5. Western Blot

For Western blot analysis, SKBR3 and BT474 cells were lysed in ice-cold buffer containing 20 mM HEPES (pH 7.4), 150 mM NaCl, 10% glycerol, and 1% Triton X-100, supplemented with Complete™ protease inhibitor cocktail, PhosSTOP™ phosphatase inhibitors, and sodium orthovanadate (all from Roche Applied Science, Penzberg, Germany). Lysis of 2D monolayers was performed directly on the plate, whereas 3D spheroids were collected and centrifuged at 375× *g* for 5 min at 4 °C prior to lysis. Lysates were incubated on ice for 30 min and clarified by centrifugation at 14,000× *g* for 10 min at 4 °C. Protein concentration was determined using the BCA assay (Thermo Fisher Scientific, Waltham, MA, USA). Equal amounts of total protein (20–30 µg) were resolved by SDS-PAGE on 4–12% Bis-Tris gels (Thermo Fisher Scientific) and transferred to 0.2 µm nitrocellulose membranes (GE Healthcare, Amersham, UK) using wet transfer at 100 V for 1 h at 4 °C. Membranes were blocked for 1 h at room temperature in 5% non-fat dry milk in TBS-T (20 mM Tris-HCl, 150 mM NaCl, 0.1% Tween-20) and incubated overnight at 4 °C with the following primary antibodies: HER2 (anti-HER2 N-terminal, Ab-20, Thermo Fisher), phospho-HER2 (Y1248; #2247, Cell Signaling Technology, Danvers, MA, USA), pan-AKT (clone 40D4, #2920, CST), phospho-AKT1/2/3 (Ser473, D9E, #4060, CST), ERK1/2 (MK1, sc-135900, Santa Cruz Biotechnology, Dallas, TX, USA), phospho-ERK (#9101, CST), and β-actin (#4967, CST). After washing, membranes were incubated for 1 h with HRP-conjugated secondary antibodies (1:5000; Jackson ImmunoResearch, West Grove, PA, USA). Signals were detected using Clarity™ ECL Western Blotting Substrate (Bio-Rad, Hercules, CA, USA) and visualized on a Uvitec Cambridge gel documentation system (UVITEC, Unit 3.05, St John’s Innovation Centre, Cowley Rd, Cambridge, UK). Band intensities were quantified using 1.47p version of ImageJ software; values were normalized to β-actin and expressed as mean ± SD from three independent experiments.

### 2.6. Statistical Analysis

All in vitro experiments were repeated at least three times. The data were analyzed using GraphPad Prism 8 (GraphPad Software, San Diego, CA, USA). Statistical differences in the average among two or more groups were compared using Student t, Mann–Whitney test, or Analysis of Variance (ANOVA), * *p* < 0.05, ** *p* < 0.01, *** *p* < 0.001, **** *p* < 0.0001.

## 3. Results

### 3.1. Distinct Morphologies and 3D Organization of HER2+ Breast Cancer Spheroids

**Figure 1:**
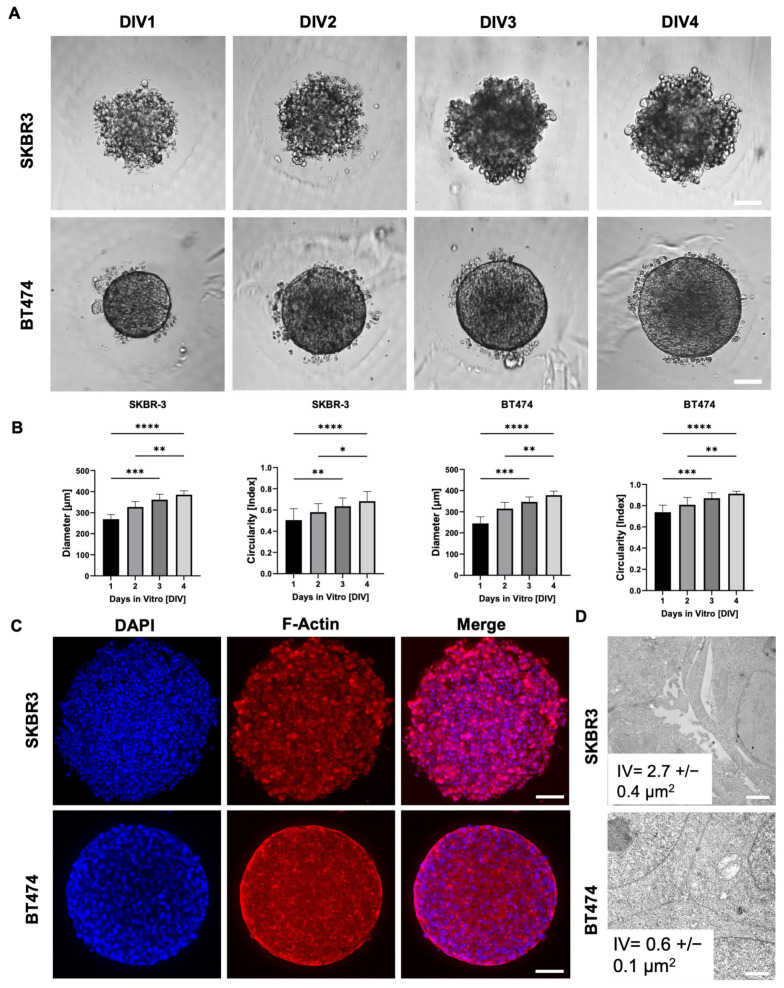
SKBR3 and BT474 spheroid formation and progression: (**A**) Representative bright-field spheroid images monitored at 1, 2, 3, and 4 days in vitro (DIV), respectively. Scale bar: 100 μm. (**B**) Spheroid size and shape parameters development over 4 days in culture: external diameter and circularity. Statistical significance is calculated by Mann–Whitney test, * *p* < 0.05, ** *p* < 0.01, *** *p* < 0.001, **** *p* < 0.0001. n = 12 for all conditions. (**C**) Representative optical images of SKBR3 and BT474 spheroids acquired with Leica Thunder microscope and labeled with phalloidin-546 (red) to detect actin cytoskeleton. In blue are depicted nuclei stained with DAPI. Scale Bar: 100 μm. (**D**) Representative electron micrographs of SKBR3 and BT474 cells showing ultrastructural details of connections among cells in 3D culture. Note that SKBR3 cells show evident interstitial spaces (interstitial voids, IV) compared to BT474.

To evaluate spheroid formation efficiency and morphological consistency, we monitored SKBR3 and BT474 spheroids over four days at four different initial cell seeding densities. Both cell lines reproducibly formed spheroids, with a progressive size increase over time (Figure 1A,B). Notably, BT474 spheroids were more compact than SKBR3 spheroids, reflecting intrinsic differences between the two models: SKBR3 cells typically proliferate and spread until confluence under 2D conditions, whereas BT474 cells display a strong propensity to form multilayered aggregates. We next quantified spheroid circularity as a measure of structural homogeneity (Figure 1B) [16]. SKBR3 spheroids displayed irregular shapes and greater variability in circularity during the four-day culture period. In contrast, BT474 spheroids rapidly achieved a near-perfect spherical shape (circularity ≈ 1) as early as day 1, maintaining this geometry with minimal fluctuation throughout the observation period. To further characterize spheroid architecture, we performed immunofluorescence imaging at day 4 using phalloidin staining to visualize filamentous actin (F-actin) [17]. Phalloidin staining delineated the cortical actin cytoskeleton, allowing visualization of cell shape and spatial arrangement within the spheroids. Confocal images acquired with the Leica Thunder system revealed that SKBR3 spheroids exhibited a looser architecture with visible intercellular spaces, whereas BT474 spheroids were denser and more cohesive (Figure 1C). To validate these morphological observations at higher resolution, we performed ultrastructural analysis by TEM [18]. Thirty intercellular regions were randomly measured across three spheroids per cell line. SKBR3 spheroids displayed wide intercellular voids (IVs) both in peripheral and central regions, while BT474 spheroids showed narrow spaces even at the periphery, confirming their tightly packed cellular arrangement (Figure 1D).

### 3.2. Assessment of Viability and Proliferation in HER2+ BCa Spheroids

**Figure 2:**
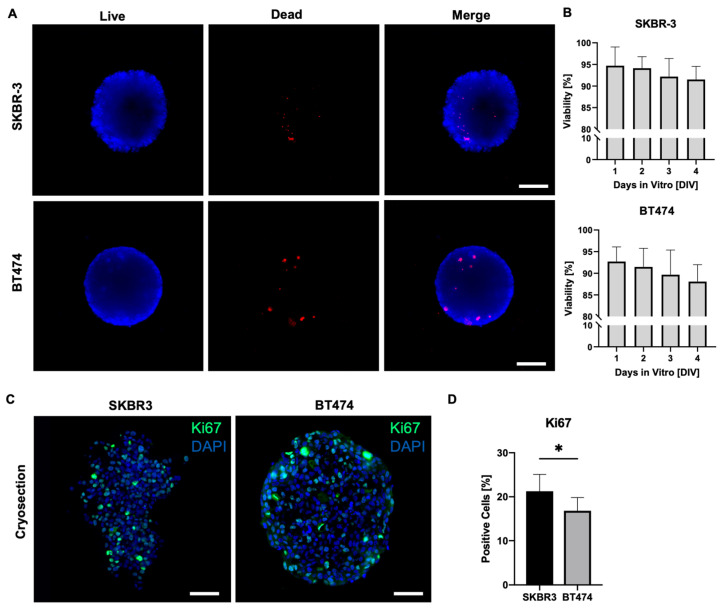
(**A**) Live/dead (blue/red, NUCLEAR-ID^®^ cell viability reagent) representative wide-field microscopy images of SKBR3 and BT474 spheroids after 4 days in culture. Scale bar: 100 μm. (**B**) Histograms showing the viability of spheroids (% of live versus dead cells) from DIV1 to DIV4. Statistical analysis is performed by ordinary one-way ANOVA test with multiple comparisons (Kruskal–Wallis test). No asterisks labeling on graphs indicates no statistically significant differences. (**C**) Representative immunofluorescence analysis of KI67 proliferation marker (green) on 5 µm thick cryosections of SKBR3 and BT474 spheroids and DAPI (blue) for nuclear staining. Images were acquired with a Leica AperioVERSA microscope. Scale bars: 100 μm. (**D**) Histograms showing the % of KI67-positive cells in spheroids of the two cell lines. Statistical significance is calculated with t test (* *p* = 0.049) (n = 6).

On day 4 of culture, we assessed cell viability in SKBR3 and BT474 spheroids using a live/dead fluorescence assay (Figure 2A) [19]. Spheroids were stained with Nuclear-ID Blue/Red and imaged using an epifluorescence microscope on a single focal plane, acquiring signals in the blue channel to detect live cells and in the red channel to detect dead cells. Merged images were processed with ImageJ to evaluate the overall viability of the outer spheroid layers. This analysis provides a two-dimensional estimate of overall spheroid viability, mainly reflecting the outer cell layers captured in the focal plane. As shown in Figure 2A,B, both SKBR3 and BT474 spheroids displayed predominantly viable outer layers, with dead cells representing < 2% of the total area across all seeding densities. These results align with previous reports showing that the peripheral zone of spheroids remains viable and proliferative during early culture stages [19,20], consistent with the observed increase in spheroid diameter over time. To overcome the intrinsic limitation of wide-field imaging and better capture proliferative activity throughout the spheroid volume, we next performed Ki67 immunofluorescence staining on spheroid cryosections [21]. Quantification of Ki67-positive nuclei revealed a significantly lower number of proliferating cells in BT474 spheroids compared with SKBR3 spheroids (Figure 2C,D) [22]. Qualitatively, BT474 spheroids displayed a clearer peripheral pattern, with Ki67 signal concentrated at the rim and markedly reduced in the inner regions. In contrast, SKBR3 spheroids exhibited a more heterogeneous distribution of Ki67-positive nuclei, without a consistently defined peripheral-to-core gradient. Ki67 expression analysis in 2D monolayer cultures confirmed that both BT474 and SKBR3 cells exhibit comparable proliferation indices under standard conditions. Quantification of confocal images revealed no significant difference in the percentage of Ki67-positive nuclei between the two lines (Figure S1), indicating that the lower Ki67 index observed in 3D spheroids reflects architecture-dependent effects rather than intrinsic differences in proliferation rate.

### 3.3. HER2 Distribution, Signaling, and Epithelial/EMT Markers in 3D Spheroids

**Figure 3:**
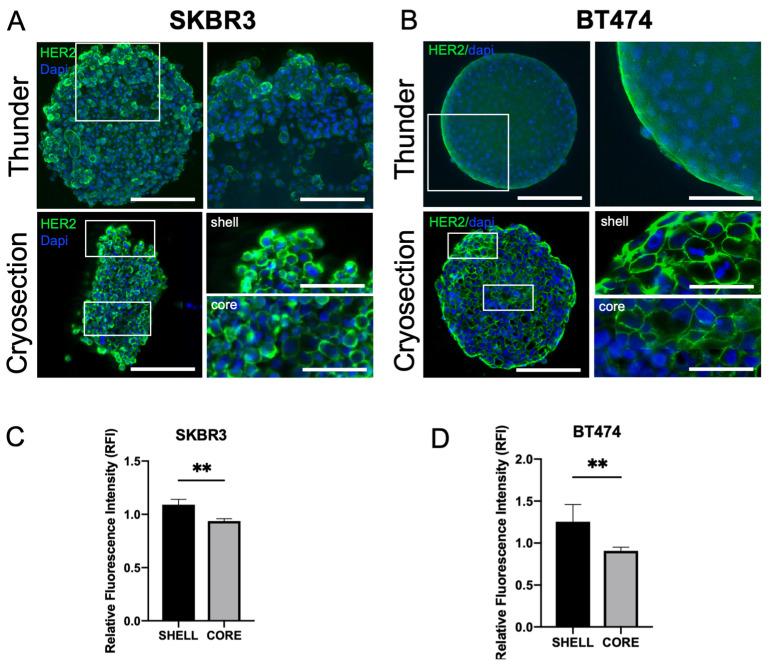
Spatial distribution and expression levels of HER2 in 3D spheroids. Confocal imaging of HER2 localization in SKBR3 and BT474 cells cultured as 3D spheroids at DIV4 ((**A**,**B**) upper panel). White boxes indicate a detail of the spheroid that has been enlarged in the right image of the panel. Images are acquired using the Thunder Leica DMi8 system. Immunofluorescence on 5 µm thick cryosections of 3D spheroids at DIV4 acquired with AperioVersa system ((**A**,**B**) lower panel). White boxes indicate two details of the spheroid that have been enlarged in the right image of the panel. Scale bars: 200 μm, insets: 50 μm. (**C**,**D**). Quantitative analysis of HER2 fluorescence intensity across spheroid regions on cryosections (shell vs. core), showing a significant decrease from the peripheral shell toward the inner core in both SKBR3 and BT474 spheroids. Statistical analysis is performed by *t*-Student test, ** *p* < 0.01. (n = 6).

HER2 expression was consistently detected at the plasma membrane in both SKBR3 and BT474 cells under 3D culture conditions [23]. In 3D, confocal imaging of whole spheroids acquired with the Leica Thunder system revealed stronger HER2 signal at the periphery compared with the inner regions (Figure 3A,B, top panels). To better visualize intratumoral gradients, we performed immunofluorescence staining on spheroid cryosections, which confirmed a progressive decrease In HER2 signal from the peripheral shell to the core (Figure 3A,B, bottom panels). Quantitative fluorescence intensity profiling across spheroid regions confirmed the significant reduction in HER2 signal in the core compared to the periphery for both SKBR3 (Figure 3C) and BT474 (Figure 3D) spheroids.

**Figure 4:**
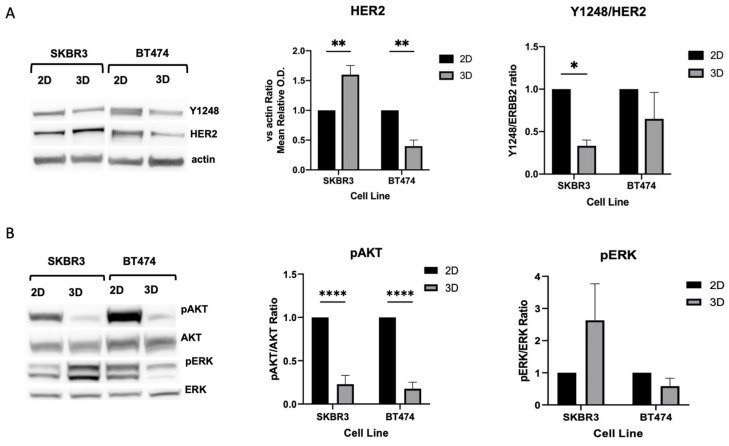
(**A**,**B**) Biochemical analysis of HER2 downstream signaling pathways. Western blot analysis comparing HER2, phosphorylated HER2-Y1248, pAKT, and pERK levels in 2D versus 3D cultures. B-actin was used as loading control. n = 3 independent experiments. Statistical analysis is performed by *t*-Student test, * *p* < 0.05, ** *p* < 0.01, **** *p* < 0.0001. No asterisks labeling on graphs indicates no statistically significant differences.

Next, to determine whether these differences reflected actual changes in protein abundance, we performed Western blot analysis of HER2 and downstream signaling components (Figure 4A). SKBR3 spheroids showed a significant increase in total HER2 protein levels compared with 2D cultures but displayed reduced phosphorylation of AKT and HER2-Y1248. Conversely, BT474 spheroids exhibited a marked decrease in HER2 protein levels compared with 2D cultures, together with lower pAKT levels and HER2-Y1248. In both 2D and 3D models, ERK phosphorylation remained unchanged (Figure 4B). Taken together, these results indicate that 3D architecture differentially regulates HER2 expression and downstream signaling across HER2+ BCa cell lines. In SKBR3 spheroids, HER2 accumulates at the membrane but is less active, suggesting altered receptor dynamics or impaired signaling competence in 3D. In contrast, BT474 spheroids show both reduced HER2 abundance and diminished AKT activity, which may reflect the stronger compaction and reduced proliferative state of this model [24].

**Figure 5:**
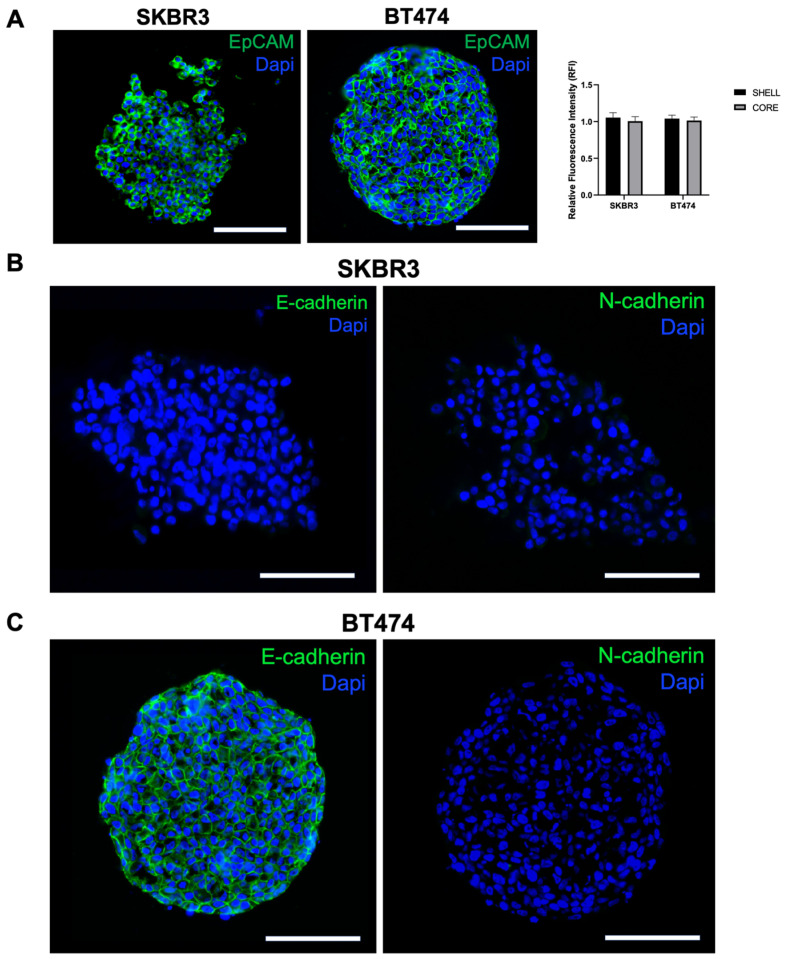
Analysis of epithelial and EMT markers in SKBR3 and BT474 spheroids: (**A**) Immunofluorescence and quantitative analysis of EpCAM signal between spheroid shell and core on 5 µm thick cryosections of 3D spheroids at DIV4. No statistical differences are observed (*t*-Student’s test, *p* = 0.207 SKBR3; *p* = 0.458 BT474). No asterisks labeling on graphs indicates no statistically significant differences. n = 6. (**B**,**C**) E-cadherin and N-cadherin immunofluorescence in the two spheroid models. Scale bars: 200 μm.

To further investigate spheroid organization and EMT characteristics, we performed immunofluorescence staining for EpCAM, E-cadherin, and N-cadherin on cryosections. EpCAM was uniformly expressed in both SKBR3 and BT474 spheroids [25], with comparable intensity in peripheral and core regions (Figure 5A, left panel and graph). In contrast, cadherin expression displayed a clear cell line-specific pattern: SKBR3 spheroids were negative for both E- and N-cadherin (Figure 5B), whereas BT474 spheroids expressed E-cadherin but lacked N-cadherin (Figure 5C). This pattern is consistent with their known phenotype in two-dimensional culture, where BT474 cells are E-cadherin-positive and SKBR3 cells are E-cadherin-negative (Figure S2) [26]. The presence of E-cadherin in BT474 is consistent with their compact, cohesive epithelial morphology, while the absence of cadherin expression in SKBR3 may underlie their looser and less organized spheroid architecture [27].

### 3.4. Ultrastructural Organization and Mitochondrial Remodeling in HER2+ BCa 2D/3D Models

**Figure 6:**
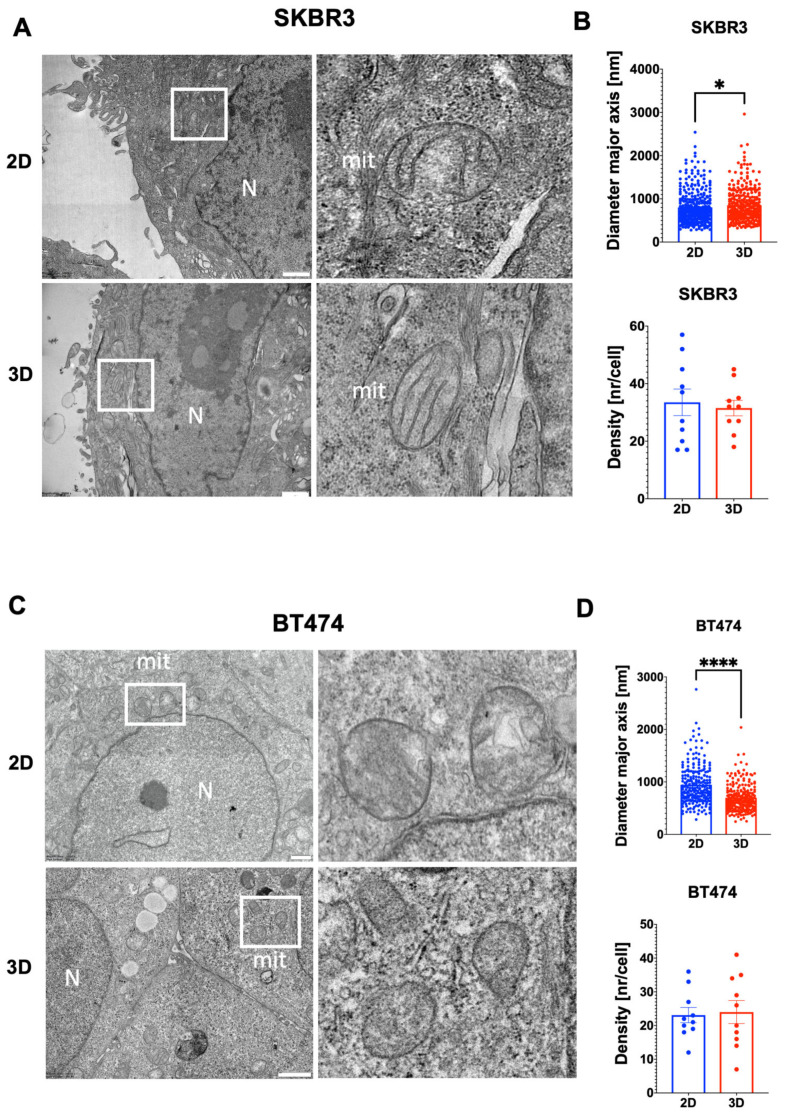
(**A**,**C**) Representative TEM images showing general cell organization and mitochondrial morphology in 2D and spheroids of SKBR3 and BT474 cells at day 4. White boxes indicate mitochondria showed in the right images of the panel. (**B**,**D**) Histograms showing the quantification of mitochondrial major axis comparing 2D and 3D spheroid showing a significant reduction in 3D versus 2D conditions for BT474 cells (**** *p* = 0.0001, Mann–Whitney test), and a slight but significant increase for SKBR3 cells (* *p* = 0.03, Mann–Whitney test). The quantification of mitochondrial density (number of mitochondria per cell) does not show any statistical difference between culture conditions (n = 3). Scale bars: 1 µm.

To investigate the ultrastructural organization of spheroids, we performed TEM analysis on SKBR3 and BT474 cells grown in 2D monolayers or as 3D spheroids. TEM imaging provided high-resolution visualization of cellular architecture in spheroids, revealing preserved overall cell morphology and the absence of central necrosis after 4 days in both models (Figure 6A,C). Mitochondria were evaluated as early indicators of subcellular adaptation to 3D culture, given their sensitivity to changes in oxygen and nutrient availability [28]. A general trend toward reduced mitochondrial number per cell section was observed in 3D for both cell lines, although not statistically significant (Figure 6B,D). Quantitative analysis showed a significant decrease in mitochondrial size in BT474 spheroids compared with 2D cultures, whereas SKBR3 cells displayed a slight but significant increase (Figure 6B–D). Qualitatively, mitochondria from BT474 spheroids appeared more disorganized than those in 2D cultures, suggesting an altered metabolic or structural state in the 3D context (6C).

## 4. Discussion

Three-dimensional spheroid cultures offer an intermediate level of complexity between 2D monolayers and in vivo tumor models, enabling a more physiologically relevant assessment of cancer cell organization, signaling, and therapeutic response [29,30,31]. In this study, we systematically characterized and compared spheroids generated from two HER2-positive cell lines, SKBR3 and BT474, using an integrated approach combining wide-field and confocal imaging, cryosection-based immunofluorescence, biochemical analysis, and ultrastructural TEM. As previously reported by Froehlich et al. [11], our results highlight pronounced differences between the two models. SKBR3 cells formed loose, irregularly shaped spheroids with heterogeneous Ki67 distribution, whereas BT474 cells generated compact, highly spherical aggregates with a well-defined proliferative rim and reduced Ki67 positivity overall. These findings indicate that the reduced Ki67 index observed in spheroids likely arises from the spatial and mechanical constraints imposed by 3D organization rather than from intrinsic differences in cell cycle regulation between BT474 and SKBR3 cells. These findings are consistent with previous reports highlighting intrinsic differences in growth patterns and cell–cell adhesion between these lines and confirm the reproducibility of our spheroid-generation approach, previously applied by our group to a spheroid model of NSC-34 cells [13]. In particular, our immunofluorescence analysis revealed that EpCAM was expressed in both models, confirming their epithelial identity, but only BT474 spheroids expressed the epithelial marker E-cadherin, whereas SKBR3 were negative for both E- and N-cadherins. This observation is in line with prior reports linking E-cadherin to tight spheroid cohesion and helps explain the more compact architecture of BT474 spheroids compared with the looser SKBR3 aggregates [32]. Consistently, the lack of E-cadherin in SKBR3 and its preservation in BT474, in both monolayer and 3D, confirms that the observed organization in spheroids mirrors their inherent adhesion phenotype [26]. Loss of cadherin-mediated adhesion in SKBR3 may also contribute to their greater intercellular spacing observed by TEM and to the heterogeneity in Ki67 distribution. In line with this epithelial divergence, previous studies have described that BT474 cells generally maintain higher expression of epithelial junctional markers, such as β-catenin and ZO-1, and lower EMT-associated regulator levels compared with more adhesion-impaired breast cancer models. Although direct comparisons in SKBR3 are fewer, the reduced E-cadherin expression and looser architecture we observed are consistent with a more mesenchymal-like phenotype [33,34,35].

By combining cryosection immunofluorescence with quantitative profiling, we demonstrated a progressive decrease in HER2 signal from the spheroid periphery to the core in both cell lines, indicating architecture-dependent receptor distribution. Western blot analysis revealed a cell line–specific response to 3D culture: SKBR3 spheroids showed increased total HER2 levels but reduced phosphorylation of HER2-Y1248 and AKT, suggesting receptor accumulation without full activation. Conversely, BT474 spheroids displayed reduced HER2 expression together with suppressed pAKT, consistent with a less proliferative, compact state. These observations align with the notion that 3D structure modulates receptor dynamics and signaling, potentially affecting therapeutic susceptibility [24,36,37]. To better contextualize our biochemical findings, it is also important to consider how 3D architecture reshapes HER2 trafficking and signaling output [38,39]. The progressive reduction in HER2 fluorescence from the periphery to the core suggests that spatial organization limits receptor accessibility and membrane turnover, in line with previous evidence that HER family receptors are highly sensitive to spatial cues and membrane compartmentalization [40]. These architectural constraints suggest a mechanistic explanation for the reduced phosphorylation of AKT and ERK observed in both lines, indicating that the 3D environment dampens PI3K–AKT and MAPK signaling independently of overall HER2 abundance. The opposite directionality of total HER2 levels in SKBR3 (increased) and BT474 (reduced) further suggests that HER2 accumulation does not necessarily translate into sustained activation in a spatially restricted context. Instead, the lower pAKT and pERK levels in 3D likely reflect altered receptor recycling, or limited internalization dynamics, all of which have been reported in compact epithelial 3D systems. Taken together, these observations support the view that 3D organization modulates not only cell–cell adhesion and epithelial polarity but also the efficiency with which HER2 couples to downstream signaling pathways. Accordingly, Ultrastructural analysis further revealed structural remodeling in 3D cultures, particularly in BT474 spheroids, which displayed smaller and less electron-dense mitochondria compared with 2D cultures. Such changes likely represent early metabolic adaptations to the hypoxic and nutrient-limited conditions of dense spheroids, as described in other 3D tumor models [39]. Notably, central necrosis was absent at day 4, supporting the use of this early time point for molecular and imaging studies without major confounding effects of cell death. Taken together, the coordinated changes in HER2 localization, AKT/ERK activity, epithelial and ETM markers, as well as mitochondria organization observed in 3D cultures, underscore how spatial organization itself reshapes oncogenic signaling, recapitulating tumor-relevant features that are not captured in 2D monolayers.

Although both SKBR3 and BT474 are long-established, immortalized cell lines that may accumulate mutations over time, they remain among the most extensively characterized HER2+ breast cancer models and capture distinct biological subtypes. Previous transcriptomic and proteomic studies have reported stable differences in epithelial and adhesion markers between these lines, consistent with our observations of divergent spheroid architecture and signaling [7,39]. Moreover, while immortalized models inherently lack the stromal and immune complexity of tumors, we show that the adoption of 3D organization restores key spatial features and diffusion constraints that more closely approximate in vivo behavior. Thus, our findings should be interpreted as an experimentally meaningful model to dissect architecture-dependent regulation of HER2 expression and signaling.

## 5. Conclusions

Collectively, our data demonstrate that 3D architecture amplifies intrinsic biological differences between HER2+ BCa cell lines, influencing cell–cell organization, receptor distribution, and downstream signaling. These results highlight the need to integrate morphological readouts with molecular analyses to fully capture architecture-dependent effects [41]. In addition, our 3D systems provide a robust and reproducible platform to investigate receptor dynamics, drug penetration and trafficking, and therapeutic responses to HER2-targeted agents in a physiologically relevant context [42].

## Data Availability

All data generated or analyzed during this study are included in this published article and its Supplementary Information files.

## Supplementary Materials

The following supporting information can be downloaded at https://www.mdpi.com/article/10.3390/biology14121654/s1, Figure S1: Representative immunofluorescence analysis of KI67 proliferation marker (red) and DAPI (blue) for nuclear staining on 2D standard monolayer cultures of SKBR3 and BT474 cells. Images were acquired with a Nikon AXR confocal microscope. Scale bars: 20 μm. (C) Histograms showing the % of KI67-positive cells of the two cell lines. No statistical significance was observed between cell lines (Mann–Whitney test, *p* = 0.155); Figure S2: E-cadherin expression in 2D monolayer cultures of BT474 and SKBR3 cells. Representative confocal immunofluorescence images showing E-cadherin (green) and nuclei (blue, DAPI) in BT474 and SKBR3 monolayer cultures. Images were acquired with a Nikon AXR confocal microscope. BT474 cells display strong membrane-associated E-cadherin, whereas SKBR3 cells are negative, mirroring the pattern observed in 3D spheroids. Images are representative of three independent experiments. Scale bar = 20 μm. Figure S3. Western blot membrane of beta actin (~48 kDa) protein detected with anti-actin (loading control) antibody (#4967, Cell Signaling, Danvers, MA, USA). Figure S4. Western blot membrane of HER2 protein detected with anti-HER2 N-terminal antibody (Ab-20, Thermo Scientific Inc. Waltham, MA, USA). Figure S5. Western blot membrane of Phospho-HER2 protein detected with anti-phosphoHER2 (Y1248) antibody (#2247, Cell Signaling Technology, Danvers, MA, USA). Figure S6. Western blot membrane of pAKT and pERK proteins detected with anti-pAKT and anti-pERK antibodies (pERK1/2 (#9101), p-AKT1/2/3 (SER473, D9E, 4060), were purchased from Cell Signaling technology (Danvers, MA, USA). Figure S7. Western blot membrane of AKT and ERK total proteins detected with anti-panAKT and anti-ERK antibodies ERK1/2 (MK1, sc-135900, Santa Cruz Biotechnology), pan-AKT (clone 40D4, #2920, Cell Signalling).

## Abbreviations

The following abbreviations are used in this manuscript:

2D	Two-dimensional
3D	Three-dimensional
ADC	Antibody–drug conjugate
AKT	Protein kinase B
BCa	Breast cancer
BT474	Human HER2+ breast cancer cell line BT-474
DAPI	4′,6-diamidino-2-phenylindole
DMEM	Dulbecco’s Modified Eagle Medium
DIV	Days in vitro
ECL	Enhanced chemiluminescence
E-cadherin	Epithelial cadherin
EMT	Epithelial–mesenchymal transition
EpCAM	Epithelial cell adhesion molecule
ERBB2/HER2	Human epidermal growth factor receptor 2
ERK	Extracellular signal-regulated kinase
F-actin	Filamentous actin
FBS	Fetal bovine serum
FCS	Fetal calf serum
IVs	Intercellular voids
Ki67	Marker of proliferation Ki-67
NH_4_Cl	Ammonium chloride
O.C.T.	Optimal cutting temperature compound
ON	Overnight
PBS	Phosphate-buffered saline
PFA	Paraformaldehyde
PVDF	Polyvinylidene difluoride
RT	Room temperature
SKBR3	Human HER2+ breast cancer cell line SK-BR-3
SDS-PAGE	Sodium dodecyl sulfate–polyacrylamide gel electrophoresis
TEM	Transmission electron microscopy
T-DXd	Trastuzumab–deruxtecan
UV	Ultraviolet
*w*/*v*	Weight/volume
