# Supplementary material for: 3D Breast Cancer Spheroids Reveal Architecture-Dependent HER2 Expression and Signaling
Source: Biology (Basel). 2025 Nov 24;14(12):1654. doi: 10.3390/biology14121654 (PMC12729668; doi:10.3390/biology14121654)
Supplement: Supplementary file 1 [file biology-14-01654-s001.zip › biology-3945915-supplementary/Supplementary file WB and FigS1-S2.pdf]

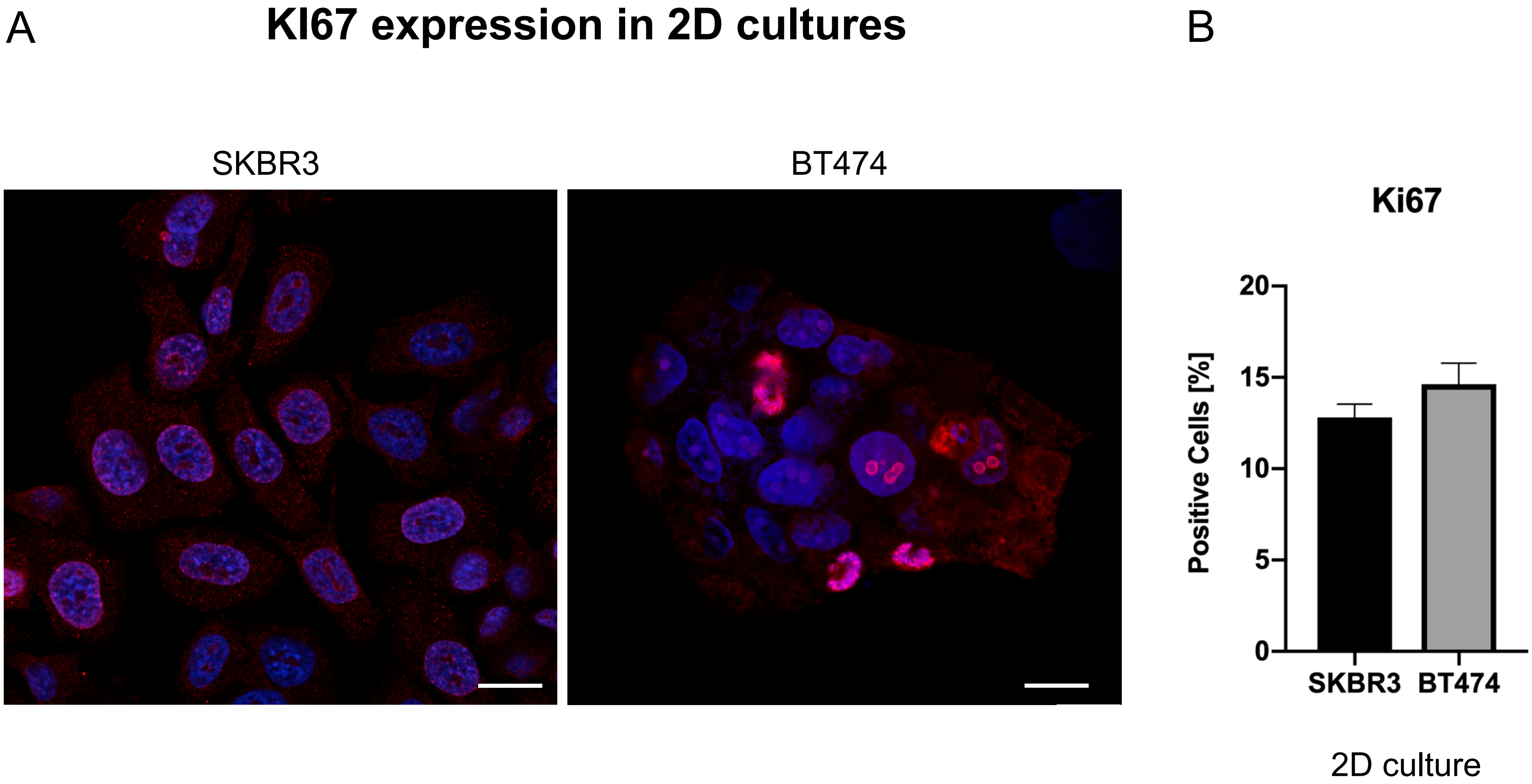
A
KI67 expression in 2D cultures
SKBR3
BT474
B
Ki67
Positive Cells [%]
20
15
10
5
0
SKBR3
BT474
2D culture

Figure S1

E-cadherin expression in 2D cultures

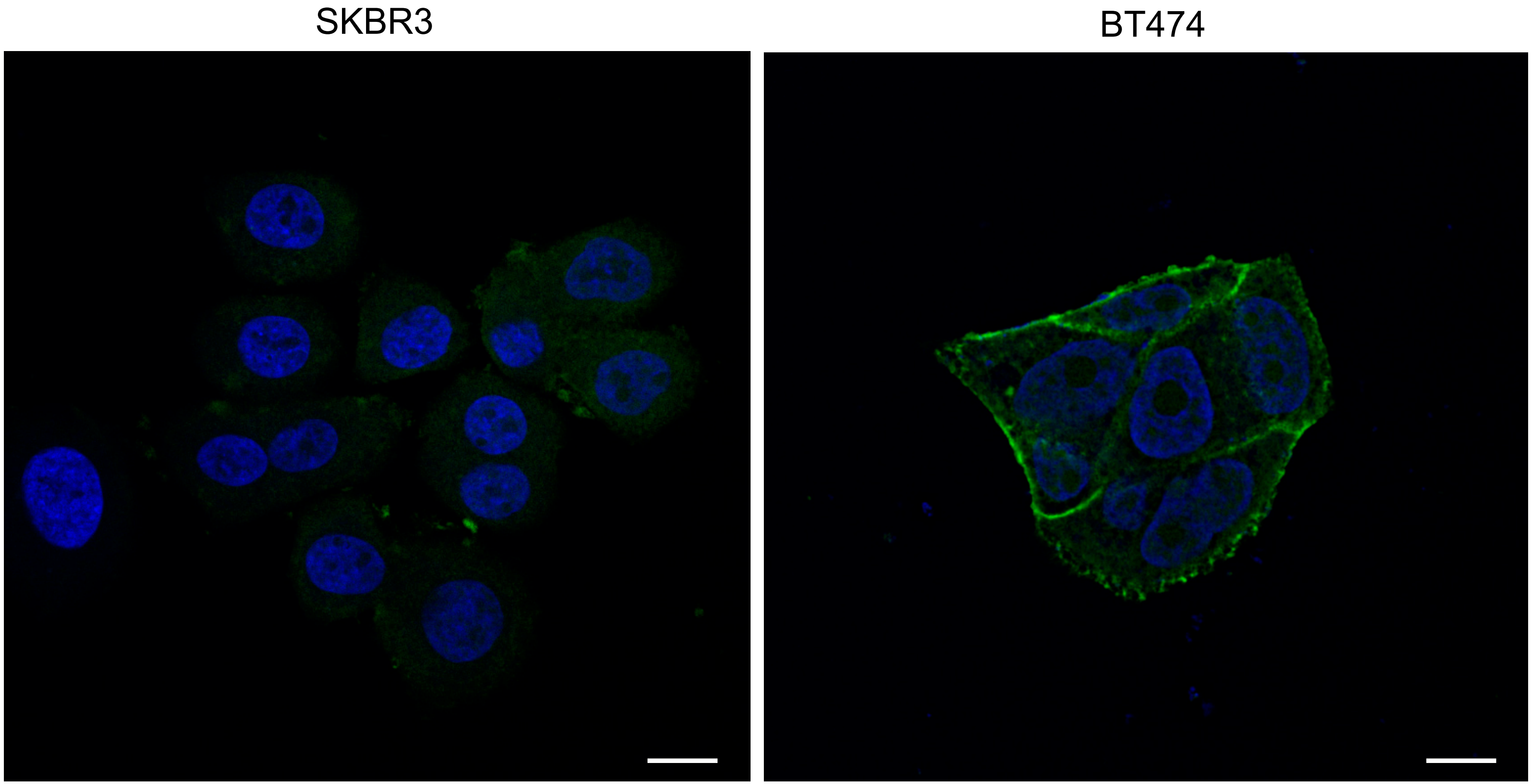

Figure S2

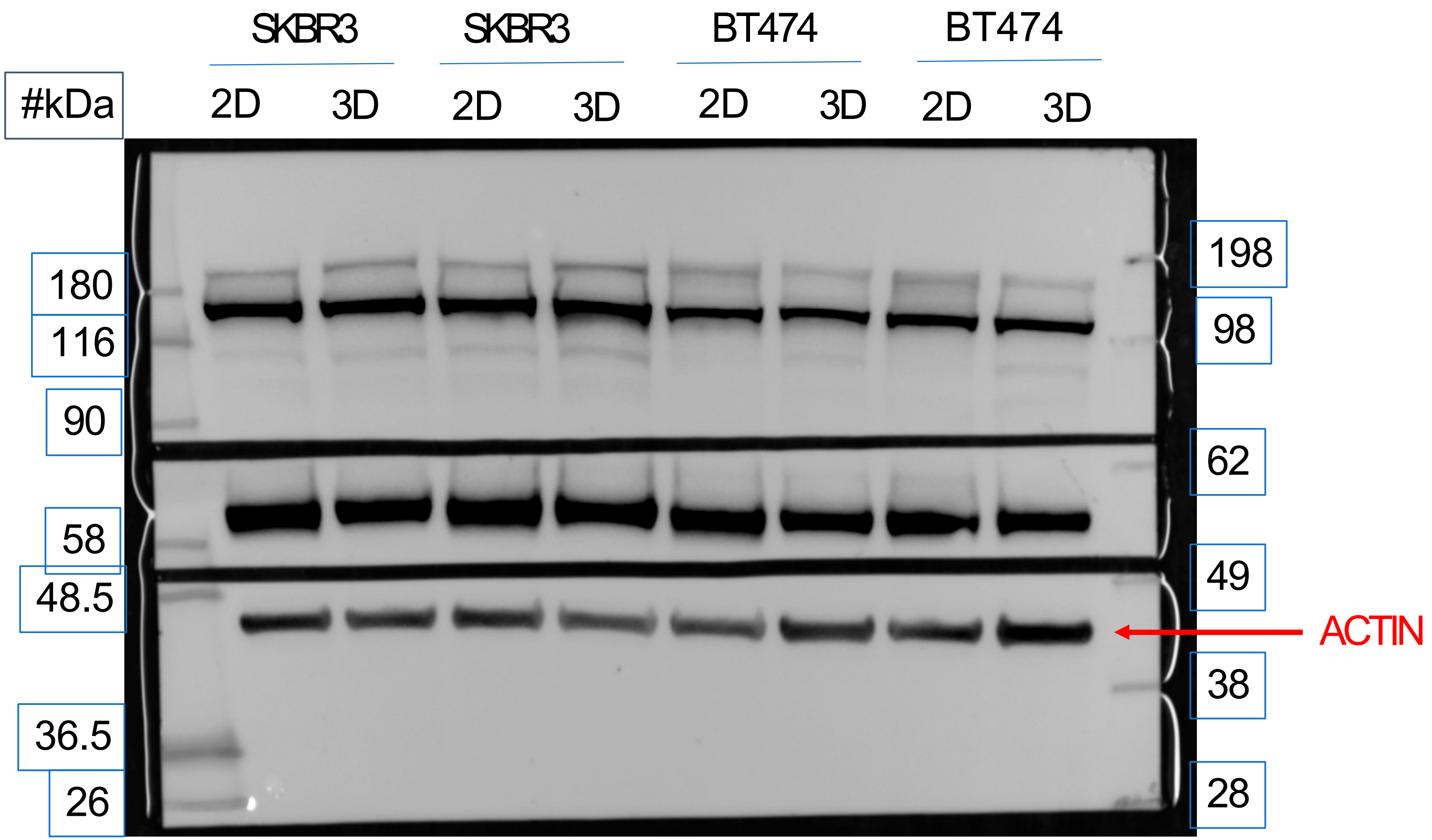

Figure S3. Western blot membrane of beta actin (~48 kDa) protein detected with anti-actin (loading control) antibody (#4967, Cell Signaling, Danvers, MA, USA). Gel-separated proteins were transferred to nitrocellulose membranes (0.2 µm pore size; Life technologies, USA) by wet electroblotting (12.7 mA per cm2, 90 min). Membranes, incubated with a horseradish peroxidase-conjugated secondary antibody (AS09 602; 1:5000–1:10000; Invitrogen), were developed with ECL Detection Reagent (BIORAD, Hercules, CA, USA). #Weight markers (molecular weight in kDa): Sigma-Aldrich Prestained Molecular weight markers (left site of the blot), 26.6 to 180 kDa; catalogue number: SDS7B2. Right side of the blot: See blue Plus2 prestained protein standard, 3 to 198 kDa, Life technologies; catalogue number: LC5925. Blot images were acquired in grayscale and then subjected to densitometry analysis using ImageJ (ImageJ v.1.49, National Institutes of Health, Maryland,USA) as follows: Image -> Adjust–> Brightness/Contrast –> Auto.

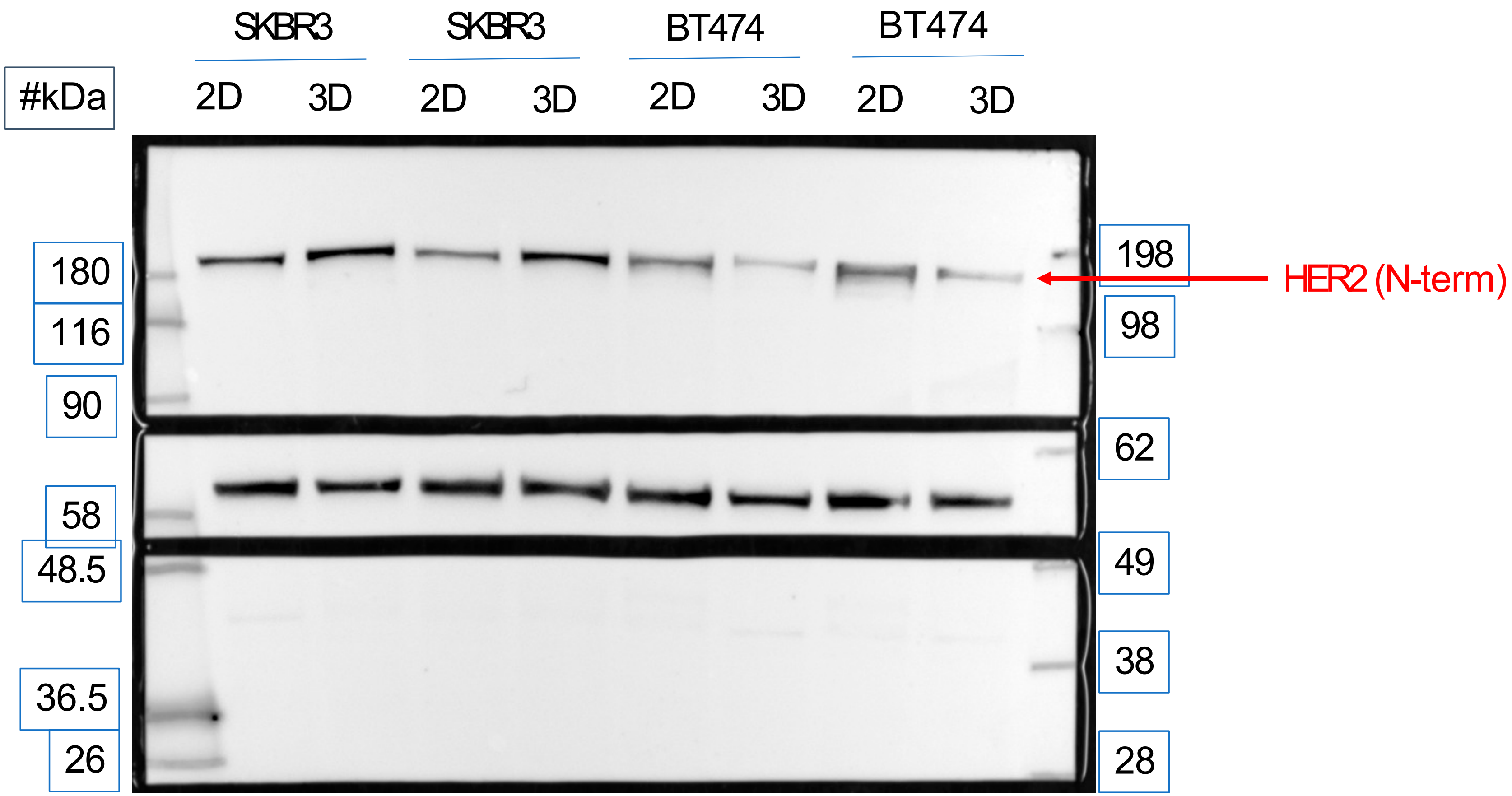

Figure S4. Western blot membrane of HER2 protein detected with anti-HER2 N-terminal antibody (Ab-20, Thermo Scientific Inc. Waltham, MA, USA). Gel-separated proteins were transferred to nitrocellulose membranes (0.2 µm pore size; Life technologies, USA) by wet electroblotting (12.7 mA per cm2, 90 min). Membranes, incubated with a horseradish peroxidase-conjugated secondary antibody (AS09 602; 1:5000–1:10000; Invitrogen), were developed with ECL Detection Reagent (BIORAD, Hercules, CA, USA). #Weight markers (molecular weight in kDa): Sigma-Aldrich Prestained Molecular weight markers (left site of the blot), 26.6 to 180 kDa; catalogue number: SDS7B2. Right side of the blot: See blue Plus2 prestained protein standard, 3 to 198 kDa, Life technologies; catalogue number: LC5925. Blot images were acquired in grayscale and then subjected to densitometry analysis using ImageJ (ImageJ v.1.49, National Institutes of Health, Maryland,USA) as follows: Image -> Adjust–> Brightness/Contrast –> Auto.

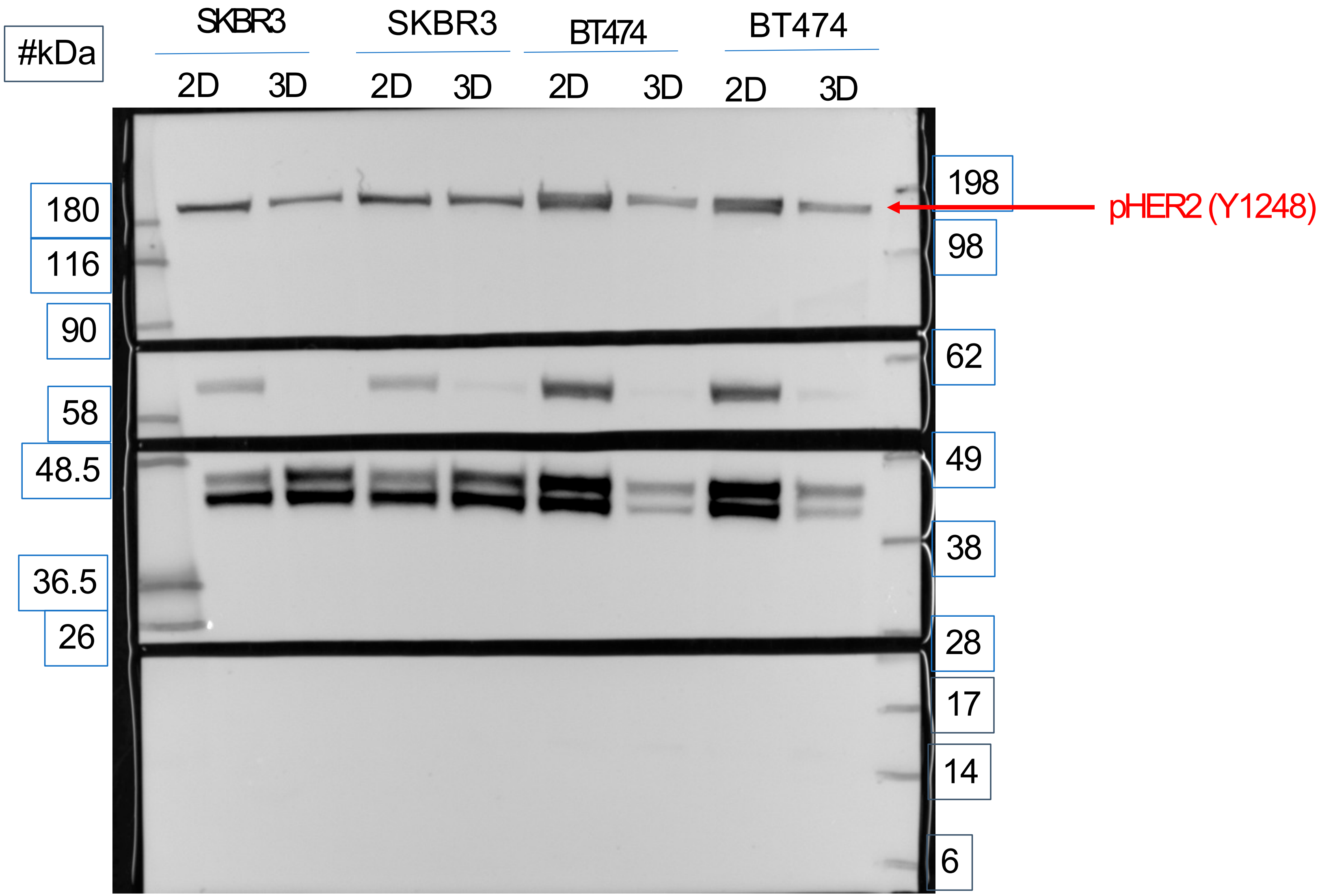

Figure S5. Western blot membrane of Phospho-HER2 protein detected with anti-phosphoHER2 (Y1248) antibody (#2247, Cell Signaling Technology, Danvers, MA, USA). Gel-separated proteins were transferred to nitrocellulose membranes (0.2 µm pore size; Life technologies, USA) by wet electroblotting (12.7 mA per cm2, 90 min). Membranes, incubated with a horseradish peroxidase-conjugated secondary antibody (G21234; 1:5000; Invitrogen, Thermo Fisher Scientific), were developed with ECL Detection Reagent (BIORAD, Hercules, CA, USA). #Weight markers (molecular weight in kDa): Sigma-Aldrich Prestained Molecular weight markers (left site of the blot), 26.6 to 180 kDa; catalogue number: SDS7B2. Right side of the blot: See blue Plus2 prestained protein standard, 3 to 198 kDa, Life technologies; catalogue number: LC5925. Blot images were acquired in grayscale and then subjected to densitometry analysis using ImageJ (ImageJ v.1.49, National Institutes of Health, Maryland,USA) as follows: Image -> Adjust–> Brightness/Contrast –> Auto.

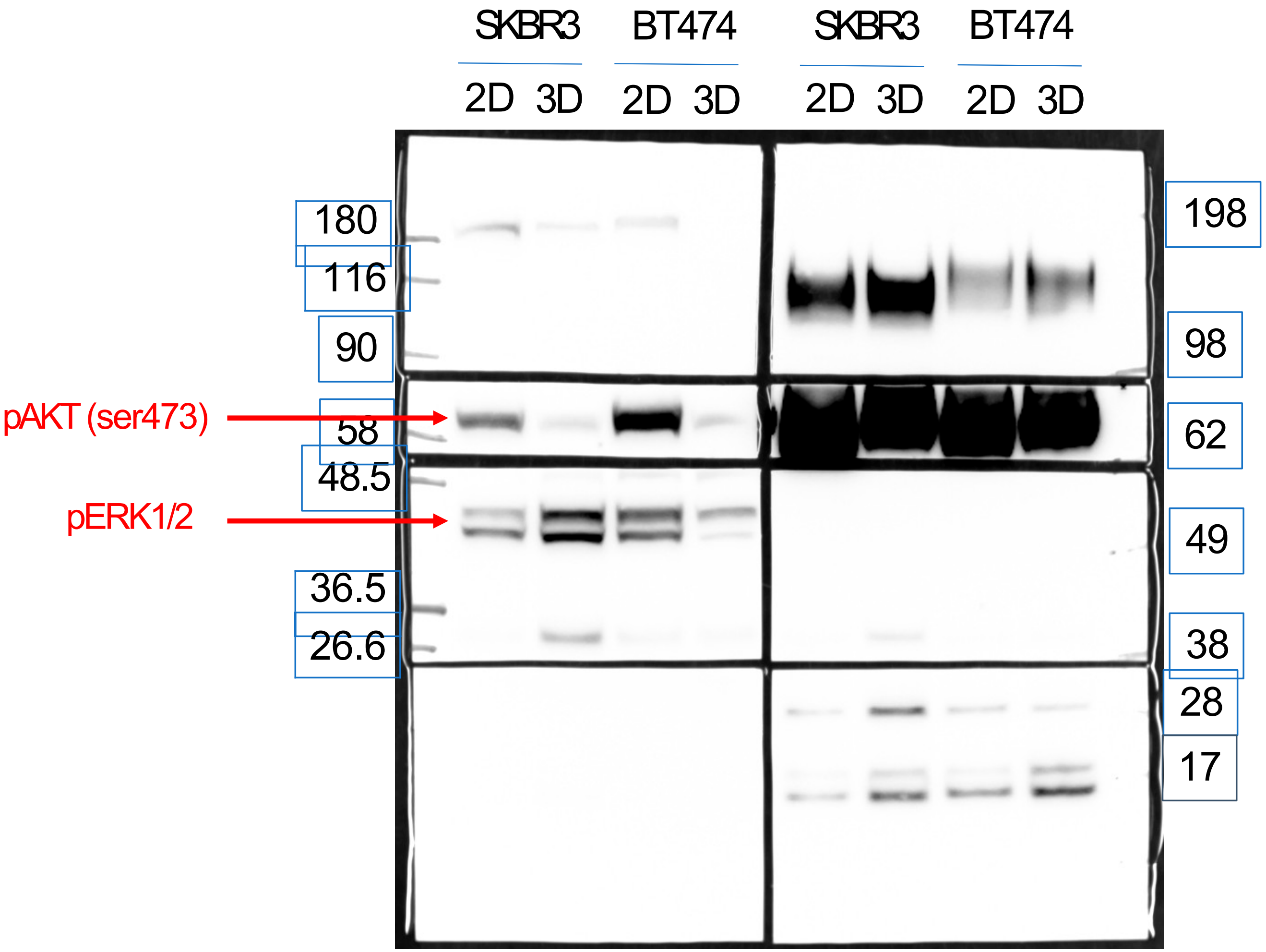

Figure S6. Western blot membrane of pAKT and pERK proteins detected with anti-pAKT and anti-pERK antibodies (pERK1/2 (#9101), p-AKT1/2/3 (SER473, D9E, 4060), were purchased from Cell Signaling technology (Danvers, MA, USA). Gel-separated proteins were transferred to nitrocellulose membranes (0.2 µm pore size; Life technologies, USA) by wet electroblotting (12.7 mA per cm2, 90 min). Membranes, incubated with a horseradish peroxidase-conjugated secondary antibody (G21234; 1:5000; Invitrogen, Thermo Fisher Scientific), were developed with ECL Detection Reagent (BIORAD, Hercules, CA, USA). #Weight markers (molecular weight in kDa): Sigma-Aldrich Prestained Molecular weight markers (left site of the blot), 26.6 to 180 kDa; catalogue number: SDS7B2. Right side of the blot: See blue Plus2 prestained protein standard, 3 to 198 kDa, Life technologies; catalogue number: LC5925. Blot images were acquired in grayscale and then subjected to densitometry analysis using ImageJ (ImageJ v.1.49, National Institutes of Health, Maryland,USA) as follows: Image -> Adjust–> Brightness/Contrast –> Auto.

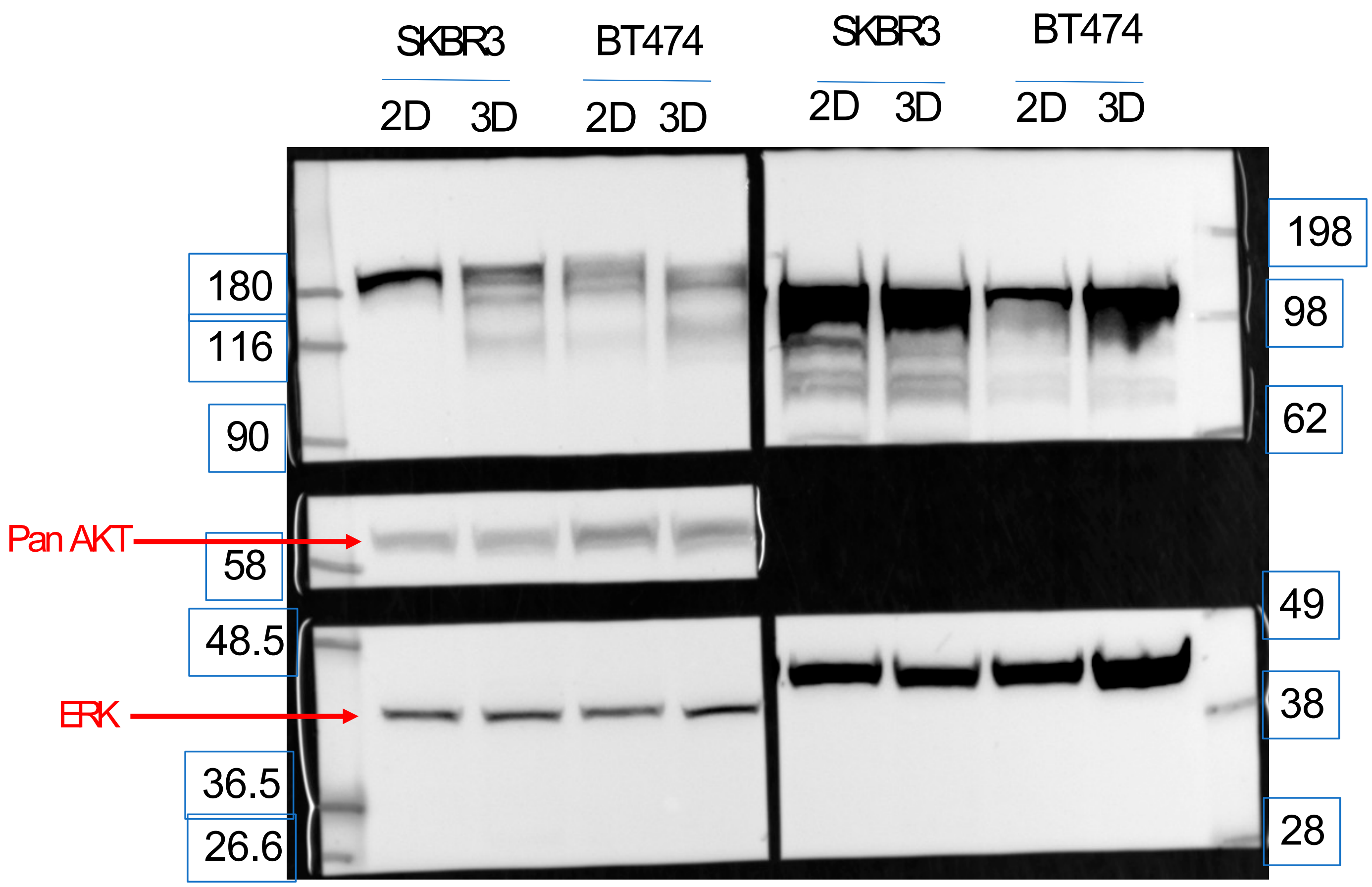

Figure S7. Western blot membrane of AKT and ERK total proteins detected with anti-panAKT and anti-ERK antibodies ERK1/2 (MK1, sc-135900, Santa Cruz Biotechnology), pan-AKT (clone 40D4, #2920, Cell Signalling). Gel-separated proteins were transferred to nitrocellulose membranes (0.2 µm pore size; Life technologies, USA) by wet electroblotting (12.7 mA per cm2, 90 min). Membranes, incubated with a horseradish peroxidase-conjugated secondary antibody (G21234; 1:5000; Invitrogen, Thermo Fisher Scientific), were developed with ECL Detection Reagent (BIORAD, Hercules, CA, USA). #Weight markers (molecular weight in kDa): Sigma-Aldrich Prestained Molecular weight markers (left site of the blot), 26.6 to 180 kDa; catalogue number: SDS7B2. Right side of the blot: See blue Plus2 prestained protein standard, 3 to 198 kDa, Life technologies; catalogue number: LC5925. Blot images were acquired in grayscale and then subjected to densitometry analysis using ImageJ (ImageJ v.1.49, National Institutes of Health, Maryland,USA) as follows: Image -> Adjust–> Brightness/Contrast –> Auto.
